# Surface Functionalization of 3D-Printed Bio-Inspired Scaffolds for Biomedical Applications: A Review

**DOI:** 10.3390/biomimetics9110703

**Published:** 2024-11-16

**Authors:** Yeon Soo Kim, Yoo Seob Shin

**Affiliations:** 1 Department of Otorhinolaryngology, Korea University Anam Hospital, Korea University College of Medicine, Seoul 02481, Republic of Korea; ionskim@gmail.com; 2 Department of Otolaryngology, Ajou University Hospital, Ajou University College of Medicine, Suwon 16499, Republic of Korea

**Keywords:** 3D printing, biomedical application, surface functionalization

## Abstract

Three-dimensional (3D) printing is a highly effective scaffold manufacturing technique that may revolutionize tissue engineering and regenerative medicine. The use of scaffolds, along with growth factors and cells, remains among the most promising approaches to organ regeneration. However, the applications of hard 3D-printed scaffolds may be limited by their poor surface properties, which play a crucial role in cell recruitment and infiltration, tissue–scaffold integration, and anti-inflammatory properties. However, various prerequisites must be met before 3D-printed scaffolds can be applied clinically to the human body. Consequently, various attempts have been made to modify the surfaces, porosities, and mechanical properties of these scaffolds. Techniques that involve the chemical and material modification of surfaces can also be applied to enhance scaffold efficacy. This review summarizes the characteristics and discusses the developmental directions of the latest 3D-printing technologies according to its intended application in unmet clinical needs.

## 1. Introduction

Three-dimensional (3D)-printing technology is a process in which structures are built layer by layer rather than by using traditional methods such as subtractive manufacturing or mixed casting [1]. Since the development of stereolithography (SLA) in 1986, which utilizes photopolymerization, various other techniques, such as fused deposition modeling (FDM) using thermoplastic filaments and selective laser sintering (SLS), have been commercialized. By 2014, with the expiration of key patents connected to early 3D-printing technologies, it became more available to the public and greatly influenced a wide range of industries, including aerospace, automotive, maritime, construction, and, most importantly, medical technology [2]. It was particularly transformative in tissue engineering. Since the 2000s, FDM, which involves melting biocompatible polymer filaments and then layering them to create structures, can be utilized for fabricating scaffolds for regenerative medicine and tissue engineering, particularly for hard biological tissues such as bone and teeth. However, for soft tissue applications, other techniques such as SLA or digital light processing (DLP) are more commonly used due to their higher resolution and smoother surface finishes. In addition to scaffolds designed for tissue engineering and cellular attachment, 3D-printing technology is actively used in various clinical settings, including surgical simulation, guide and implant production, and the creation of patient-customized prosthetics [3]. The ability of 3D printing to rapidly and efficiently produce complex 3D biomimetic structures from a variety of biocompatible materials underpins its growing utilization in numerous medical applications. In this paper, ‘biomaterials’ are defined as biocompatible and potentially biodegradable materials designed to interact with biological systems, supporting cell adhesion, growth, differentiation, and fulfilling specific physiological roles in biomedical applications.

Through millions of years of evolution, nature has optimized various biological structures to achieve a delicate balance of efficiency, strength, and flexibility, each of which is tailored for specific functions. Natural structures such as the bones, skin, and spiderwebs possess intricate and sophisticated microstructures that are challenging to replicate through artificial design. By harnessing these bio-inspired structures, we can leverage nature’s optimized design principles to develop artificial constructs that demonstrate superior performance. Thus, bio-inspired scaffolds have been engineered to emulate the physical, chemical, and mechanical properties of human tissues and organs. These characteristics are particularly crucial in tissue engineering and regenerative medicine, areas in which biomaterials must interact with the human body while maintaining biocompatibility. By copying natural forms and functions, bio-inspired structures can promote cell adhesion, growth, and differentiation while minimizing immune responses, thereby enhancing implantation success rates. A key advantage of bio-inspired scaffolds is their ability to offer multifunctionality beyond that of single-purpose designs. For instance, structures inspired by nature can simultaneously provide strength and flexibility or maintain moisture retention while preserving breathability. This multifunctionality is invaluable for a range of biomedical applications including medical devices, tissue scaffolds, and drug delivery systems. The versatility of bio-inspired structures is particularly important when addressing complex biological environments that require multiple functionalities to overcome the limitations of conventional artificial constructs.

Three-dimensional printing has emerged as a powerful tool for realizing bio-inspired designs. The advent of advanced 3D-printing technologies has made it possible to create complex customized scaffolds that closely mimic natural structures. This synergy between bio-inspiration and 3D printing opens new possibilities for creating scaffolds with precise geometries, specified porosities, and tailored mechanical properties. The application of 3D-printing technology in clinical medicine can be broadly classified into four main production categories: (1) precise anatomical models for surgical simulation or training, aiding medical education; (2) surgical guides and instruments used to improve surgical resection precision and safety (e.g., mandibular resection guides and fibular flap harvesting guides); (3) external medical devices (e.g., prosthetic limbs, artificial ears, and radiotherapy fixation masks); and (4) implantable medical devices designed to replace or support missing tissue or organ function [4]. Medical devices in categories (1), (2), and (3), which do not have direct contact with and are not implanted into the body (Class I–III devices), do not necessarily require biomaterials and are relatively less challenging to produce. However, category (4) (Class IV) medical devices, which are implanted into the body and carry a high potential risk, must be manufactured precisely using safe biomaterials.

Food and Drug Administration (FDA)-approved polymers and metallic biomaterials must be used in compliance with Good Manufacturing Practice standards. These materials must be modified and improved prior to 3D production to prevent the elicitation of immune responses [5]. Furthermore, when applying 3D-printing technology to create scaffolds for regenerative medicine, using biocompatible, naturally derived materials that can incorporate with living cells to form the required organ structure can potentially increase the rates of integration with existing tissues and cells while reducing rejection and immune responses compared with the use of synthetic scaffolds. Consequently, the research and development of 3D-printing technologies utilizing various biomaterials are being actively pursued.

Bio-inspired 3D-printed scaffolds offer unique opportunities for surface functionalization. Modifying the surface properties of these scaffolds can enhance their biocompatibility, cellular interactions, and functional performance. By combining the structural advantages of bio-inspired designs with strategic surface modifications, we can create scaffolds that mimic natural structures and possess additional functionalities tailored to specific biomedical applications.

This comprehensive review overviews the characteristics of 3D-printing technologies aimed at enhancing the biocompatibility of various biological and synthetic materials. Here, we investigate the surface treatments of scaffolds and explore the latest advancements in attachment techniques that incorporate functional factors such as growth factors, antibodies, and peptides. These cutting-edge 3D-printing technologies represent the forefront of biomedical engineering and offer unprecedented control over scaffold architecture and surface properties. By critically analyzing these developments, we aim to provide insight into the future directions of 3D-printed bio-inspired scaffolds and their potential to revolutionize tissue engineering, regenerative medicine, and other biomedical applications.

## 2. Print Manufacturing Technology

Three-dimensional-printing manufacturing technology fundamentally relies on the principle of layer-by-layer deposition to create 3D structures. The techniques involved can be categorized based on the resolution and accuracy of the reproduction of the 3D structure, the methods used to achieve these, and the materials employed in 3D printing. Each technique has its own set of advantages and disadvantages (Table 1). Following the development of SLA, several other 3D-printing technologies were developed and commercialized, including FDM, SLS, DLP, color jet printing, and multijet printing [6,7,8]. These technologies have been widely adopted in various fields, including medical and industrial applications, in which precision, material flexibility, and application-specific performance are critical.

FDM, currently the most widely used 3D-printing technology, melts or extrudes biocompatible polymer materials to fabricate solid 3D structures. FDM is the simplest and most cost-effective 3D-printing technology, offering the broadest range of material options suitable for human applications. However, FDM has notable drawbacks including low resolution and accuracy as well as suboptimal surface quality. Moreover, the need for support structures that maintain the integrity of complex 3D shapes can limit the production of complex designs.

SLA (stereolithography) is another widely utilized technique known for its high resolution and excellent surface quality. SLA uses a laser to cure photosensitive resins layer by layer, making it particularly effective for creating detailed and intricate parts. This technology is beneficial for applications requiring complex geometries, such as dental devices and surgical tools. However, the equipment can be expensive, and the process often requires support structures, which may add to post-processing steps.

DLP, another widely used technique in the medical field, operates similarly to SLA but with faster print speeds owing to the use of a digital projector to cure an entire layer of resin simultaneously. DLP offers high-resolution and smooth surface finishes, making its use ideal for detailed applications such as dental prosthetics, hearing aids, and other intricate medical devices. However, similar to SLA, DLP requires photopolymers as the primary material, which can be expensive and may limit the number of applications owing to the brittleness of the final product. In addition, post-processing is required to remove excess resin and improve the mechanical properties of the printed parts.

Material jetting is a high-resolution 3D-printing method that allows for the creation of multimaterial and color-capable parts. This technique is highly precise and is often used for detailed prototypes and models in medical applications. The downside is its high cost and the brittle nature of the printed objects, which can limit their use in functional, load-bearing applications.

SLS is a powder-based printing technology that uses a laser to fuse particles of polymers, metals, or ceramics to create high-resolution, durable parts. It is particularly valued for its ability to produce complex geometries without the need for support structures, because the surrounding powder provides natural support during the printing process. SLS is ideal for producing highly functional medical components such as custom implants, prosthetics, and dental devices, owing to its strength, accuracy, and capacity to work with a broad range of materials. However, it requires expensive equipment and materials, and the printed parts often require post-processing, such as surface smoothing and sintering, to achieve the necessary mechanical properties and aesthetic quality.

DMLS (direct metal laser sintering) is an advanced technique that allows for the creation of high-strength metal parts and supports a wide range of materials. This method is particularly useful in producing custom medical implants and components for the aerospace industry. The major challenges include its high cost and the extensive post-processing required to achieve the necessary mechanical properties and surface finish.

DLP (digital light processing), another widely used technique in the medical field, operates similarly to SLA but with faster print speeds owing to the use of a digital projector to cure an entire layer of resin simultaneously. DLP offers high-resolution and smooth surface finishes, making its use ideal for detailed applications such as dental prosthetics, hearing aids, and other intricate medical devices. However, similar to SLA, DLP requires photopolymers as the primary material, which can be expensive and may limit the number of applications owing to the brittleness of the final product. In addition, post-processing is required to remove excess resin and improve the mechanical properties of the printed parts.

Extrusion printing of biomaterials is particularly significant in tissue engineering and regenerative medicine as it allows for the printing of living cells using biocompatible bio-inks. This technology is adaptable to various bio-inks and can create complex biological constructs that support cell growth and tissue formation. However, extrusion printing has lower resolution compared to other methods and its effectiveness depends on the mechanical stability of the printed bio-ink structures.

## 3. Bio-Inspired Design Principles in 3D Printing

Biological species exhibit diverse geometric structures and excellent mechanical properties including high stiffness, strength, durability, and resistance to wear, fatigue, and corrosion. Inspired by these geometric structures, high-performance materials and structures have been fabricated for various applications. Biomimetic technology, or bio-inspiration, is an effective approach to reimagining technology, particularly in biomedical engineering. As bio-inspired design principles are integrated into 3D-printing technologies, it is possible to develop innovative structures and materials that mimic the efficient and functional design of nature and play a significant role in fields such as medicine, tissue engineering, and artificial organ development.

Fibrous structures occur naturally in various tissues, including the muscles, tendons, bones, and skin. These structures are robust and flexible, creating an ideal environment for cell adhesion and proliferation. Bio-inspired fiber scaffolds typically comprise biocompatible and biodegradable polymer materials. Polymers such as polylactic acid (PLA), polycaprolactone (PCL), and polyglycolic acid (PGA) are widely used because of their ability to gradually degrade within the body to harmless metabolites. This degradation mechanism permits the scaffolds to spontaneously degrade over time and be replaced by newly produced tissues at their original sites.

Bio-inspired scaffolds can be developed using various methods, such as electrospinning, hydrogel and aerogel formation, and extracting cells from tissue grafts, with 3D printing being an essential technique for precisely replicating complex structures. The ability of 3D printing to replicate the fine microstructures and geometric patterns of fiber scaffolds makes it well-suited to tissue engineering applications. These structures provide an ideal environment for cellular attachment and proliferation and act as scaffolds for tissue regeneration. Bio-inspired scaffolds produced through 3D printing are used to regenerate tissues such as cartilage, bone, and skin and can be applied to the development of artificial organs. These scaffolds possess physical and mechanical properties similar to those of human tissues and promote cellular attachment and growth.

Furthermore, 3D-printing technology allows the fabrication of customized scaffolds tailored to the specific needs of individual patients. Additionally, surface-functionalization techniques can be employed to enhance the integration of cells and tissues into scaffolds, thereby ensuring improved compatibility and functionality.

The combination of bio-inspired design principles and 3D-printing technology is enabling transformative innovations in various fields, including medical implants, tissue engineering, and artificial organ development. In particular, the use of 3D printing for customized scaffolds and surface-functionalization techniques facilitates personalized treatments. As multimaterial and high-resolution printing technologies continue to advance, more complex and functional bio-inspired scaffolds are expected to be developed. Bio-inspired design is an important direction in 3D-printing technology, leading to the creation of more efficient and functional therapeutic solutions. The combination of bio-inspired design and 3D printing will play a crucial role in the future of medicine, particularly in the areas of tissue regeneration, artificial organs, and personalized medical devices.

## 4. Fabrication of 3D-Printed Scaffolds Using Biocompatible Biodegradable Polymers

Biocompatible degradable polymers used for medical purposes are a special category of polymers that decompose via hydrolysis or enzymatic degradation processes within the body into harmless natural byproducts, such as H_2_O, CO_2_, N_2_, and inorganic salts.

As FDA-approved materials, PGA, PLA, poly(lactic-co-glycolic acid), and PCL are used to produce various biodegradable sutures, screws, artificial blood vessels, skin, stents, and internal fixation devices (Table 2).

Among them, PCL has a very low glass transition temperature (−60 °C) and exhibits flexible rubber-like properties at room or body temperatures with a similarly low melting temperature (60 °C). Given these characteristics, since the early 2000s, PCL has been pre-processed into filaments through extrusion molding and printed using 3D printers. Consequently, it is among the first materials to be researched and clinically applied in the production of 3D-printed tissue engineering scaffolds [9].

In various medical applications, 3D-printed customized biodegradable products have received clinical approval and are widely used in treatments such as orbital fracture repair, skull and maxillofacial reconstructions, alveolar bone regeneration, and plastic surgery [10]. Moreover, research has increasingly focused on single biocompatible polymers as well as composite materials incorporating substances such as tricalcium phosphate and hydroxyapatite [11]. For instance, PLA-hydroxyapatite (HA) composites have demonstrated enhanced osteoconductivity, making them particularly favorable for bone tissue regeneration. Polymer blends such as PTMC/PLA combine flexibility and strength and offer superior mechanical properties. From a processing perspective, functional heterogeneous materials can be printed using separate nozzles to enhance the functionality of the 3D-printed scaffolds. Kang et al. demonstrated the effective integration of bioprinting technology by alternating the 3D printing of PCL and a cell–hydrogel composite [12]. Park et al. reported a biomimetic scaffold fabrication technique using alternating layers of PCL-printed structures with nanofiber scaffolds that mimicked the extracellular matrix, showcasing advances in biomimetic scaffold manufacturing [11].

The technology for creating biocompatible scaffolds by manufacturing bio-inks using an extracellular matrix derived from living organisms and then 3D printing using them is also being actively researched [13]. Material extrusion is the most actively developed and applied method owing to the advantages of combining various biomaterials and the availability of low-cost printing equipment [14].

## 5. Micro/Nano Printing Techniques for Advanced Biomedical Applications

Micro/nano 3D-printing technologies, including two-photon polymerization (2PP), electrohydrodynamic (EHD) printing, and nanolithography, have made major advances in the production of complex microstructures for biomedical purposes. These methods are noted for their high precision and ability to replicate intricate biological architectures, making them essential for developing structures that closely mimic natural tissues. 2PP uses concentrated femtosecond laser pulses to reach submicron resolution, allowing for the construction of intricate 3D scaffolds that improve cell adhesion and tissue integration [15]. EHD printing, which uses electric fields to deposit material from fine nozzles, creates nanofibrous scaffolds that are analogous to the extracellular matrix, promoting cell adhesion and proliferation [16]. Nanolithography also permits the development of nanopatterned surfaces that can guide cell behavior and adherence, which is crucial for designing biomaterials customized to specific biological responses [17].

Micro/nano-scale 3D-printing techniques have significantly advanced biomedical applications by enabling the fabrication of scaffolds with controlled porosity and microstructures, which support cellular growth and tissue regeneration. These methods also enhance drug delivery systems through precise control over release profiles, improving therapeutic efficacy and reducing side effects. Customized implants and medical devices produced at these scales better align with patient-specific anatomy, leading to improved integration and performance. However, challenges such as high production costs, technical complexity, and scalability hinder widespread clinical adoption. Ensuring reproducibility and consistency in the printing process is essential for reliable biomedical applications. Future advancements should focus on developing cost-effective, scalable methods and versatile materials, including bio-inks that mimic the properties of natural tissues. Integrating living cells directly into printed microstructures holds promise for accelerating tissue regeneration and advancing personalized medicine, paving the way for next-generation medical solutions tailored to complex biological needs.

## 6. Surface Functionalization for Biocompatible Scaffolds

**Figure 1:**
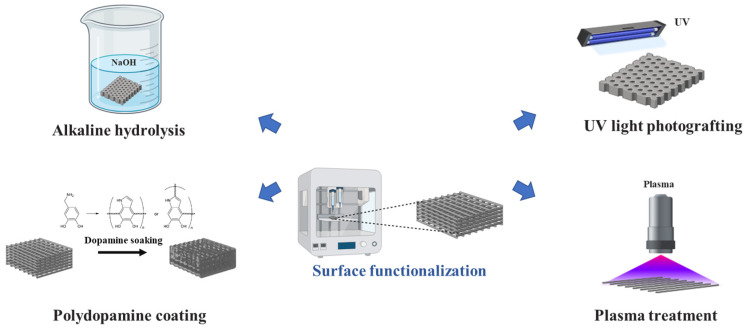
Comparison of different surface-functionalization methods. UV, ultraviolet.

Surface functionalization involves coating 3D-printed scaffolds with various functional materials, including biologically active molecules such as growth factors, antibodies, peptides, nucleic acids, and their derivatives. This technique modifies the scaffold surface through chemical or physical treatments such as alkaline hydrolysis, ultraviolet photografting, and plasma treatment to enhance the scaffold’s biocompatibility. Various surface-functionalization methods are shown in Figure 1, while their principles, advantages, and disadvantages are listed in Table 3.

Surface functionalization is crucial to ensuring that 3D-printed scaffolds not only provide structural support but also actively contribute to the healing process. These methods improve the wettability of materials and enhance their ability to interact with biological tissues, which is particularly beneficial for medical applications. Functional materials include biocompatible or chemically active coatings that promote interactions between 3D-printed components and biological entities [18].

### 6.1. Surface Modification Using Alkaline Hydrolysis

During alkaline hydrolysis, the alkaline solution reacts with the surface of the 3D-printed scaffold material, breaking down ester bonds and creating carboxylate anions (–COO–) and hydroxyl (–OH) groups on the surface [19]. The surface erosion mechanism involves the removal of the upper layer of the 3D-printed scaffold surface, thereby exposing carboxylate groups. These carboxylate groups enhance the scaffold’s interaction with functional groups in the solution, improving surface wettability, adhesion, printability, and dyeability [20]. This method uses strong bases such as sodium hydroxide (NaOH) to improve the surface properties of the scaffolds. When the scaffold is immersed in an NaOH solution, the ester and nitrile groups on the scaffold are hydrolyzed, resulting in the formation of carboxylic acid and amide groups, which confer greater polarity and hydrophilicity than the original surface. As the concentration of the NaOH solution and treatment time increase, the hydrophilicity and surface roughness significantly improve. This increased roughness allows better integration with surrounding tissues, promoting the formation of a strong tissue–implant interface. Improved hydrophilicity facilitates protein adsorption, which is essential for the early stages of cellular attachment and proliferation. By adjusting the NaOH solution concentration and treatment duration, the degree of hydrolysis can be controlled, allowing the scaffold’s surface properties (e.g., adhesion, wettability, and biocompatibility) to be modified to suit specific applications and purposes. This is one of the simplest and most cost-effective surface treatments. However, as the hydrolyzed products may include various chemicals, such as residual monomers or byproducts from the degradation of the polymer matrix depending on the exact composition of the 3D-printed material and the reaction conditions, caution is required during hydrolysis.

### 6.2. Polydopamine Coating

Polydopamine (PDA), an adhesive protein derived from mussels, is a bio-inspired surface-modification technique that has been widely applied in chemistry, biology, medicine, and materials science because of its unique surface-coating ability and abundance of active sites [21]. Over the past decade, extensive research has been conducted on the physicochemical properties of PDA, including its biocompatibility, biodegradability, metal ion chelation, redox activity, and other potentially useful characteristics. Among these attractive features, adhesive properties and chemical reactivity have been actively used to activate the surfaces of 3D-printed scaffolds.

Dopamine, particularly in alkaline solutions, can form thin films of adhesive PDA on various material surfaces via oxidative polymerization. These PDA films provide an essential platform for secondary reactions by binding reactive groups, such as catechol, amines, and imines, to the surface, serving as a starting point for covalent bonding with a wide variety of desired molecules. PDA nanomembrane roughness also influences adhesion, making PDA coatings crucial for the surface functionalization of 3D-printed scaffolds through various mechanisms as mentioned above [22].

Although the self-polymerization of dopamine and deposition of PDA are relatively straightforward, the process is time-consuming. It typically involves the dissolution and polymerization of dopamine monomers (dopamine hydrochloride) in an alkaline aqueous solution (commonly Tris-HCl buffer at pH 8.5) over a period of >12 h, during which the solution changes from colorless to dark brown [23].

PDA-coated PCL scaffolds reportedly exhibit improved surface hydrophilicity, making them more conducive to cellular attachment and proliferation. PDA-coated surfaces can be further functionalized with bioactive molecules such as growth factors, proteins, and peptides to promote specific biological responses. However, PDA coatings have some limitations, including a lack of electrical conductivity, which restricts their use in applications such as nerve tissue engineering and bioelectronics. In addition, controlling the coating thickness is challenging and the coating may degrade over time, potentially affecting the scaffold’s long-term stability and performance.

Future research on PDA coatings should focus on improving control over their thicknesses and exploring new functionalization strategies to enhance the biological activity of 3D-printed scaffolds. Additionally, combining PDA with other surface-modification techniques, such as plasma treatment or ultraviolet (UV) light photografting, could lead to synergistic effects that further enhance scaffold performance in biomedical applications.

### 6.3. UV Light Photografting

UV light photografting is a surface-modification technique that uses UV light to graft functional groups onto a material’s surface. UV light can initiate a wide range of polymerization reactions, creating covalent bonds that connect functional agents to the material surface. Photografting on substrates improves hydrophilicity and biocompatibility by allowing the grafting of hydrophilic functional groups onto the surface [24].

When exposed to UV radiation, the photoinitiator in the binding array generates free radicals that trigger the formation of covalent bonds between the binding monomers and the substrate’s surface. A photoinitiator is a substance that forms radicals when exposed to UV or visible light, facilitating the efficient initiation of the crosslinking reaction of polymers or ligands, which are photo-crosslinking materials. Monomer types and concentrations that cause covalent bonding, exposure time, and UV light intensity comprise various factors that affect surface modifications. Photografting can also increase surface roughness, which may enhance the mechanical bonding of functional agents. By binding various materials to a substrate via photografting, enhanced hydrophilicity and biocompatibility can improve cellular penetration into the substrate and reduce immunogenicity [25]. It also reduces the processing steps, time, and costs compared with other methods that require surface pre-activation. Despite its benefits, UV light photografting can modify only the surface layer, making its use less effective for scaffolds with complex internal structures. Additionally, this technique is effective only for certain polymers that can generate free radicals under UV light exposure, restricting its use to a narrow range of materials.

### 6.4. Plasma Treatment

Plasma treatment, a widely used technique for the surface functionalization of 3D-printed scaffolds, involves modifying material surfaces using non-thermal atmospheric plasma, which interacts with the scaffold’s surface to introduce reactive groups or modify the surface topography.

Upon exposure to various plasma types, including oxygen, nitrogen, and argon, physicochemical modifications occur on the substrate’s surface that can improve surface hydrophilicity, cellular adhesion, and biodegradability [26]. Plasma treatment can also add various functional groups, including carboxylic acids, hydroxyl groups, and amino groups, to the substrate’s surface. These functional groups can increase the surface’s energy and hydrophilicity, thereby improving its adhesion to other materials.

Interactions between the plasma, which includes ions, radicals, and electrons, and surface functional groups of the substrate can create new chemical bonds and functional groups. The plasma-induced etching of the substrate surface results in physical alterations. The uppermost layer of the substrate’s surface can be removed via plasma treatment, revealing the underlying surface and potentially enhancing its characteristics. Non-thermal atmospheric plasma can improve substrate properties, such as cellular adhesion, hydrophilicity, durability, and immunogenicity, while being environmentally friendly owing to its minimal chemical use. This versatile and noninvasive method is applicable to various materials, such as polymers, metals, and ceramics, without altering their bulk properties. Moreover, plasma treatment is highly customizable, allowing precise control over parameters such as gas type, pressure, and treatment time to achieve tailored surface modifications. Despite its advantages, plasma treatment requires specialized and costly equipment and modifies only the surface layer of the scaffold, limiting its efficacy for complex structures. In addition, the process is highly sensitive to treatment conditions, with small variations in the parameters significantly affecting the results.

## 7. Benefits of Surface Functionalization for Biocompatible Scaffolds

### 7.1. Wettability Control

Surface functionalization can adjust the wettability of 3D-printed surfaces, making them hydrophilic or hydrophobic as required [27]. This property is crucial for biomedical devices, microfluidics, antifouling applications, and oil–water separation systems. Techniques such as plasma treatment, dip coating, and self-assembly are commonly employed to modify surface wettability.

### 7.2. Enhanced Biocompatibility

Improving biocompatibility in 3D-printed medical devices and scaffolds is essential for their successful integration with human tissues. Methods such as plasma treatment, coating with bioactive materials such as hydroxyapatite, and incorporating biological factors such as collagen and growth factors promote cell adhesion, proliferation, and differentiation on the scaffold, facilitating tissue integration and regeneration.

### 7.3. Antibacterial Properties

Surface-functionalized 3D-printed devices can be modified to reduce infection risk by incorporating antibacterial agents such as silver nanoparticles, antimicrobial peptides, or zinc oxide nanorods [28]. These modifications are particularly important for medical implants, where antibacterial surfaces prevent bacterial adhesion and kill microbes upon contact.

### 7.4. Metallization

Surface metallization enhances the electrical, mechanical, and thermal properties of 3D-printed structures, making them suitable for applications requiring conductivity and durability [29]. Techniques such as electroless plating and polymer-assisted metal deposition enable the creation of conductive metallic coatings on complex 3D surfaces, valuable for electronic and mechanical components.

### 7.5. Mechanical Strengthening

Surface functionalization can also improve the mechanical properties of 3D-printed components. Techniques such as ultrasonic nanocrystal surface modification and atomic layer deposition of ceramic coatings increase strength, stiffness, and wear resistance, making these parts more durable in demanding applications [19,30,31].

### 7.6. Catalytic Performance

Functionalizing 3D-printed structures with catalytic materials transforms them into effective and reusable catalysts for industrial and biocatalytic applications. Methods include incorporating metal–organic frameworks (MOFs) and immobilizing enzymes on the surface, which enable efficient catalysis for various chemical processes.

These surface modifications are essential for adapting 3D-printed devices to practical applications across biomedicine, electronics, environmental engineering, and chemical processing, enhancing their functionality and performance in specialized roles.

## 8. The Emerging Field of Bioprinting

The emerging field of bioprinting has attracted considerable attention. This technology involves the direct use of biocompatible materials, including living cells, in 3D printing to create structures and holds promise for applications related to regenerative medicine and tissue engineering [32,33]. Unlike traditional 3D-printing methods that use polymers or metals, bioprinting involves the deposition of biocompatible materials and even living cells to create complex biological structures. This approach aims to create biocompatible and implantable medical devices capable of delivering cells and drugs into the human body and potentially replacing the functions of various organs [34]. Additionally, bioprinting with biocompatible materials and human-derived cells allows the production of bio-inks that can replicate the histological structure and functionality of real organs.

A key advantage of this technology is the ability to create patient-specific tissues that can be implanted without the risk of immune rejection. One of the most promising applications of bioprinting is the development of lab-on-a-chip devices that can replicate the histological structure and functionality of real tissues and organs for drug testing and disease modeling. These devices allow the testing of drugs and other therapeutics in a controlled environment that closely mimics the human body, thereby reducing the need for animal testing and accelerating the development of new treatments [35,36]. Moreover, the ability to print functional tissues opens the door to personalized medicine, in which treatments are specifically tailored to an individual’s unique genetic and cellular makeup.

## 9. Discussion

Three-dimensional-printing technology has fostered revolutionary advances in the medical field. This technology enables the production of patient-specific implants and medical devices, enabling the accurate 3D reconstruction of complex defective tissues in individual patients. This offers new hope for the treatment of intractable diseases and facilitates precise and effective surgical planning. As clinical experience and records accumulate and are digitally managed, it is possible to manufacture medical devices for tissue regeneration using only patients’ medical images and a 3D printer, even without face-to-face consultations. This presents the potential ability to standardize and improve medical care quality both nationwide and globally.

Several practical challenges must be overcome before 3D printing can be actively and universally adopted in clinical medicine. First, image standardization and quality improvement are needed for processing and modeling based on clinical medical imaging. Second, standardized treatment guidelines for 3D-printed medical devices must be developed to reflect clinical factors that are difficult to identify through imaging alone based on diverse clinical experiences. Third, a process is required to enable the selection of appropriate image segmentation, modeling, 3D-printing technologies, and materials that correspond to clinical demands. The development of printing technologies and research on material improvement falls within the realm of translational medicine, requiring active collaboration between clinicians and researchers as well as significant research and development investment for the creation of disease- and patient-specific scaffolds. Therefore, protocols must be established and new technologies introduced to ensure that the abovementioned series of tasks can be performed rationally and efficiently in the medical field.

With advancements in medical technology, 3D printing has significantly propelled the development of medical scaffolds. Techniques that mimic natural structures for surface functionalization have been increasingly employed to enhance biocompatibility, promoting scaffold integration and compatibility with human tissue. According to Chakraborty et al., scaffold-functionalization methods are broadly divided into surface and bulk modifications, which enhance biocompatibility by altering the scaffold’s physical, chemical, and mechanical properties. Surface modifications include physical methods (e.g., plasma treatment, UV irradiation) that improve surface topography and hydrophilicity, chemical methods (e.g., wet chemical etching) that introduce functional groups such as carboxyl and hydroxyl groups to increase surface roughness, and coating methods (e.g., layer-by-layer assembly) that deposit bioactive layers such as proteins to improve cellular adhesion. Each method aims to optimize scaffold acceptability and integration within biological environments [37].

If these challenges are addressed, tissue regeneration scaffolds and implantable medical devices utilizing 3D-printing technology are likely to develop further and be widely applied in clinical medicine. This will significantly contribute to optimized treatments for individual patients and improved overall quality of medical care.

## 10. Conclusions

3D-printing technology has transformed the fabrication of bio-inspired scaffolds by allowing for accurate replication of natural structures required for tissue engineering and regenerative medicine. Surface-functionalization strategies, including as physical, chemical, and biological approaches, have proven crucial in improving the biocompatibility, mechanical strength, and usefulness of these scaffolds. These functionalization strategies promote scaffold integration with human tissues by improving surface characteristics, which aid in cellular adhesion, proliferation, and differentiation. This paper discusses how such technologies can enhance the clinical utility of 3D-printed scaffolds, making them appropriate for a wide range of biomedical applications, including tissue regeneration, implantable devices, and bioengineered organ development.

## 11. Future Directions

Despite the advances described, there are still enormous prospects for future progress in this sector. Future research should concentrate on combining multifunctional surface treatments, which will enable 3D-printed scaffolds to satisfy complicated biological requirements such as regulated drug delivery and infection prevention. Furthermore, advances in bioprinting may permit the direct inclusion of living cells within functionalized scaffolds, hastening tissue regeneration processes. Exploring innovative biomaterials, as well as sustainable and scalable manufacturing methods, will be critical for bringing these technologies into clinical use. Furthermore, standardizing functionalization procedures and regulatory frameworks is critical for assuring the safety and efficacy of bio-inspired scaffolds in tailored medicinal applications.

## Figures and Tables

**Table 1:** Various three-dimensional-printing technologies.

Printing Technology	Materials	Strengths	Limitations
Fused deposition modeling	Thermoplastics	Low cost, easy to operate, variety of usable materials	Long printing time, low print resolution, often requires supports
Stereolithography	Photosensitive resins	High resolution, excellent surface quality	Long printing time, expensive equipment, requires supports
Digital light processing	Photosensitive resins	High speed, high-quality prints	High cost, requires support structures
Material jetting	Photosensitive resins	High resolution, multimaterial properties, color capability	High cost, brittle mechanical properties, requires supports
Selective laser sintering	Powdered materials (nylon, glass, ceramic, metal)	High accuracy, large-volume building capability	High cost, difficult to operate and calibrate
Binder jetting	Powdered materials (sand, metal, ceramic)	Multi-color printing, no need for support structures	High opacity, requires post-processing
Direct metal laser sintering	Metals	High-strength metal parts, variety of material options	High cost, extensive post-processing required
Extrusion printing of biomaterials	Biocompatible hydrogels, bio-inks containing cells	Capable of printing living cells, supports tissue engineering and regenerative medicine	Lower resolution, dependent on bio-ink properties, limited by mechanical stability of structures

**Table 2:** FDA-approved biodegradable polymers for three-dimensional printing.

Abbreviated Name	Full Name	Key Characteristics	Common Applications
PLA	Polylactic acid	- FDA-approved synthetic biodegradable polymer - Derived from renewable resources, such as corn or sugarcane - Approved for food contact	- Medical implants - Drug delivery systems - Tissue engineering scaffolds
PGA	Polyglycolic acid	- FDA-approved biodegradable thermoplastic polyester - Rapid degradation rate	- Sutures - Tissue engineering - Drug delivery systems
PLGA	Polylactic-co-glycolic acid	- FDA-approved biodegradable copolymer - Tunable degradation rate	- Drug delivery systems - Tissue engineering scaffolds - Nanoparticles for medical applications
PCL	Polycaprolactone	- FDA-approved biodegradable polyester - Slow degradation rate - High drug permeability	- Long-term implants - Tissue engineering scaffolds - Drug delivery systems
PBS	Polybutylene succinate	- FDA-approved biodegradable polymer - Good thermal and mechanical properties	- Medical devices - Tissue engineering - Drug delivery systems

FDA, Food and Drug Administration.

**Table 3:** Overview of surface-modification techniques.

Technology	Principle	Advantages	Disadvantages
Alkaline hydrolysis	Alkaline solution reacts with material surface to introduce carboxyl and hydroxyl groups, increasing hydrophilicity and biocompatibility	Improves hydrophilicity; enhances biocompatibility; cost-effective and simple	May damage surface if over-processed; difficult to achieve uniform modification on large structures
Polydopamine coating	Dopamine-based coating adheres strongly to various surfaces and provides catechol and amine groups to enhance cell bioactivity	Strong adhesion to various materials; improves biocompatibility and hydrophilicity; easy functionalization	Requires precise control of coating thickness; may degrade over time affecting long-term stability
Ultraviolet (UV) light photografting	Uses UV light to graft materials such as GelMA onto scaffold surface without additional pretreatment	Reduces cost by avoiding extra chemicals; fast treatment time	Photooxidative degradation; unwanted bulk polymerization
Plasma treatment	Plasma activates material surface, increasing hydrophilicity and enhancing cell adhesion	Improves hydrophilicity and adhesion; environmentally friendly, minimal use of chemicals	Requires complex and expensive equipment; results highly sensitive to treatment conditions

GelMA, gelatin methacryloyl.

## Data Availability

Data are contained within the article.
